# Decitabine Rescues Cisplatin Resistance in Head and Neck Squamous Cell Carcinoma

**DOI:** 10.1371/journal.pone.0112880

**Published:** 2014-11-12

**Authors:** Chi T. Viet, Dongmin Dang, Stacy Achdjian, Yi Ye, Samuel G. Katz, Brian L. Schmidt

**Affiliations:** 1 Department of Oral Maxillofacial Surgery, New York University, New York, New York, United States of America; 2 Bluestone Center for Clinical Research, New York University, New York, New York, United States of America; Institute of Clinical Physiology, c/o Toscana Life Sciences Foundation, Italy

## Abstract

Cisplatin resistance in head and neck squamous cell carcinoma (HNSCC) reduces survival. In this study we hypothesized that methylation of key genes mediates cisplatin resistance. We determined whether a demethylating drug, decitabine, could augment the anti-proliferative and apoptotic effects of cisplatin on SCC-25/CP, a cisplatin-resistant tongue SCC cell line. We showed that decitabine treatment restored cisplatin sensitivity in SCC-25/CP and significantly reduced the cisplatin dose required to induce apoptosis. We then created a xenograft model with SCC-25/CP and determined that decitabine and cisplatin combination treatment resulted in significantly reduced tumor growth and mechanical allodynia compared to control. To establish a gene classifier we quantified methylation in cancer tissue of cisplatin-sensitive and cisplatin-resistant HNSCC patients. Cisplatin-sensitive and cisplatin-resistant patient tumors had distinct methylation profiles. When we quantified methylation and expression of genes in the classifier in HNSCC cells *in vitro*, we showed that decitabine treatment of cisplatin-resistant HNSCC cells reversed methylation and gene expression toward a cisplatin-sensitive profile. The study provides direct evidence that decitabine restores cisplatin sensitivity in *in vitro* and *in vivo* models of HNSCC. Combination treatment of cisplatin and decitabine significantly reduces HNSCC growth and HNSCC pain. Furthermore, gene methylation could be used as a biomarker of cisplatin-resistance.

## Introduction

More than 60% of head and neck squamous cell carcinoma (HNSCC) patients present with advanced-staged disease, which is associated with a high mortality rate [1]. The current treatment for advanced-stage HNSCC is cisplatin and radiation for patients with good performance status; patients with limited performance status receive high-dose cisplatin alone [2]–[4]. Cisplatin resistance occurs in some patients and significantly reduces survival as there are no effective alternative therapies. The mechanism of cisplatin resistance is multifactorial and poorly understood [5]. In addition, none of the known mechanisms are reversible with drug therapy.

Aside from poor survival, HNSCC patients have significantly more pain than other cancer patients [6], [7]. A meta-analysis of 52 studies evaluating prevalence of cancer pain shows that HNSCC has a higher prevalence of pain compared to all other sites [8]. HNSCC-induced pain limits orofacial functions such as swallowing, mastication and speech, which results in poor quality of life. In fact, outside of survival, pain-induced loss of function is the biggest concern for head and neck cancer patients [9], [10]. Given the severe symptoms and reduced survival of HNSCC patients, a novel pharmacologic approach that both reduces cisplatin resistance and alleviates pain is needed.

DNA methylation is an epigenetic silencing mechanism that has recently been proposed as a mechanism for cisplatin resistance [11]. Unlike other chemotherapy resistance mechanisms, DNA methylation is reversible by demethylating drugs; decitabine is one of the most potent demethylating drugs. Decitabine has been used in clinical trials for hematological and solid malignancies, with the major side effect being transient and manageable myelosuppression [12]–[15]. We showed from previous studies in a preclinical HNSCC model that decitabine not only inhibits tumor growth, it also treats pain-induced loss of function [16].

Based on our preliminary studies we hypothesize that methylation is a reversible mechanism of cisplatin-resistance. Moreover, we propose that decitabine could be added to cisplatin chemotherapy to rescue cisplatin-resistance in HNSCC and alleviate cancer-induced pain. We use both *in vitro* and preclinical models to determine the anti-tumor and analgesic effects of decitabine on cisplatin-resistant HNSCC. To identify patients at risk for cisplatin resistance and those who would benefit from decitabine, we perform methylation profiling by analyzing biopsies from HNSCC patients treated with cisplatin.

## Methods

### Patient recruitment and tissue collection

All procedures were approved by the Institutional Review Board at New York University. A waiver of informed consent was granted in accordance with 45 CFR 46.116(d). We identified patients from 2005–2010 who had 1) biopsy-proven HNSCC, 2) no history of prior surgical or chemoradiation treatment for HNSCC, and 3) cisplatin-based chemotherapy with or without radiation. We obtained formalin-fixed, paraffin embedded (FFPE) initial incisional biopsies, performed prior to chemotherapy, for each patient. All patients received a CT scan pre-treatment and six months post-treatment; tumor progression was assessed by a radiologist by comparing pre- and post-treatment scans. Progression was classified with Response Evaluation Criteria in Solid Tumors (RECIST), with RECIST 1 signifying progressive disease (PD), RECIST 2 signifying stable disease (SD), RECIST 3 signifying partial response (PR), and RECIST 4 signifying complete response (CR).

### Cell culture and drug treatments

SCC-25, a tongue SCC, and SCC-25/CP, which was made cisplatin-resistant by continuous cisplatin treatment [17], were obtained from Dr. John Lazo. The cells were cultured in Dulbecco's Modified Eagle Medium (DMEM), supplemented with 10% fetal bovine serum (FBS). For decitabine treatment, SCC-25 and SCC-25/CP were plated at 25% confluence on 10 cm plates and treated with 5 µM freshly-prepared decitabine (Sigma) in DMEM with supplements. Drug and media were changed every 24 hours until cells were confluent. Decitabine-treated cells were subsequently referred to as DAC-SCC-25 or DAC-SCC-25/CP.

### Proliferation and apoptosis assays

SCC-25, SCC-25/CP, DAC-SCC-25, and DAC-SCC-25/CP were plated in 96-well plates at a density of 5,000 cells/well. Cells were treated with either cisplatin (1–300 µM) or drug vehicle (3% DMSO in DMEM supplemented with 2% FBS); drug and media were replenished after 24 hrs. Cell viability was quantified using the MTS assay (Promega) after 48 hours of drug treatment. Apoptosis was quantified with the Caspase-Glo-3/7 assay (Promega) after 24 hours of drug treatment.

### Cancer mouse model

The cancer pain mouse model was produced as previously described [18]. Experiments were performed on female BALB/c, athymic mice weighing 16–20 g at the time of SCC inoculation. All the procedures were approved by the New York University Committee on Animal Research. Researchers were trained under the Animal Welfare Assurance Program. 5×10^6^ SCC-25/CP cells were suspended in Matrigel (Becton Dickinson & Co.) to a volume of 50 µl and inoculated into the plantar surface of the right hind paw. 2–4% isoflurane inhalational anesthesia was used for inoculation. Twenty-four mice were divided into four groups, (1) combination treatment with decitabine (6 mg/kg) and cisplatin (6 mg/kg), (2) decitabine only (6 mg/kg), (3) cisplatin only (6 mg/kg), and (4) drug-vehicle control. Decitabine was dissolved in phosphate-buffered saline (PBS), filter-sterilized, and administered intraperitoneally (IP) at a volume of 200 µl on post-inoculation days (PID) 7 and 9. Cisplatin was dissolved in PBS with 1% dimethyl sulfoxide (DMSO), filter-sterilized, and injected IP at a volume of 200 µl on PID 12, 15, 18, and 21. Based on a previous study [19] the third-day, two week duration dosing of cisplatin is optimal in controlling tumor growth and minimizing normal tissue damage.

Paw volume measurements were performed to quantify cancer growth with a plethysmometer (IITC Life Sciences) as described [18]. Paw withdrawal testing was performed to evaluate mechanical allodynia as described [18]. Testing was performed by an observer blinded to the experimental groups between 0900 and 1200 h. Paw withdrawal thresholds were determined in response to pressure from an electronic von Frey anesthesiometer (IITC Life Sciences). The amount of pressure (g) needed to produce a paw withdrawal response was measured six times on each paw separated by 3 minute intervals. On PID 30 animals were euthanized with 4% isoflurane.

### Sodium bisulfite modification and Methylight

5×10^6^ cells were harvested from culture, homogenized with a Mini Beadbeater-1 (BioSpec Products) and subject to DNA/RNA extraction with AllPrep DNA/RNA Kit (Qiagen). Five 10 µm sections of formalin-fixed, paraffin embedded tissue from patients were subject to RNA/DNA extraction with the AllPrep DNA/RNA FFPE Kit (Qiagen). Methylight probes and primers for promoter regions of *CRIP1, G0S2, MLH1, OPN3, S100* and *TUBB2A* were designed with Beacon Designer (Premier Biosoft). Sodium bisulfite conversion was performed according to manufacturer's recommendations using the EZ DNA Methylation Kit (Zymo Research). Methylight PCR was performed as previously described with *COL2A1* as the internal control gene [20]. Percentage of methylated reference (PMR, *i.e.*, degree of methylation) was calculated for each sample using M.SssI-treated, CpGenome universal methylated DNA (Millipore) as the positive control.

### Quantitative reverse transcription PCR (RT-PCR) analysis

mRNA was reverse transcribed with Random Hexamers (Applied Biosystems). A 2 µl cDNA aliquot was amplified with the Taqman gene expression assay for the gene of interest, which did not detect residual genomic DNA. PCR was also performed to detect HPV16 E6 mRNA as described [21]. Human GAPDH was used as endogenous control. Delta-delta CT was used for relative quantification.

### Statistical analysis

Statistical analysis was performed using Sigma Plot, version 11.0. Data was analyzed using Student's t-test, One-way ANOVA, Two-way ANOVA or Two-way RM ANOVA with Holm Sidak or Tukey *post hoc* testing as appropriate. Results were presented as mean ± standard error of the mean (SEM).

## Results

### Decitabine pre-treatment enhanced the cytotoxic and apoptotic effects of cisplatin on cisplatin-resistant cells

**Figure 1:**
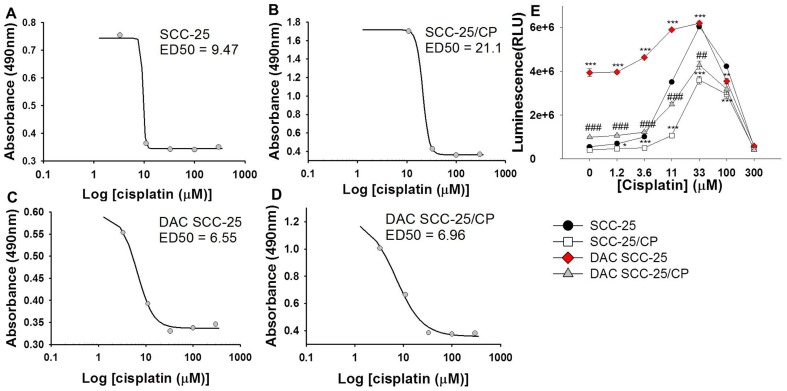
Decitabine pre-treatment enhances cytotoxic and apoptotic activity of cisplatin. (**A-D**) Cell viability is represented by absorbance (y-axis) and compared to common log of cisplatin concentration (µM). ED50, the concentration needed to inhibit viability by 50%, is listed for each cell line. (**E**) The graph depicts apoptosis activity (caspase 3/7 activity, measured as luminescence on y-axis) at different cisplatin concentrations (3-300 µM). A decrease in apoptotic activity at high dose ranges is an expected phenomenon in this assay due to early cell death from high drug doses prior to quantification. Decitabine pre-treatment in cisplatin-resistant SCC-25/CP cells increases apoptotic activity of cisplatin relative to non-treated SCC-25/CP cells. Decitabine pre-treatment in cisplatin-sensitive SCC-25 cells also increases apoptotic activity of cisplatin at lower doses. One Way ANOVA, Holm-Sidak test pairwise comparisons, *p<.05, **p<.01, ***p<.001, compared to SCC-25; #p<.05, ##p<.01, ###p<.001, compared to SCC-25/CP.

Based on the cell viability assay, we determined the effective dose-50 (ED-50) of cisplatin, which is the dose required to inhibit viability by 50%. The ED-50 of SCC-25 was 9.47 µM, whereas the ED-50 of SCC-25/CP was 21.1 µM. However, decitabine pre-treatment enhanced the cytotoxicity effect of cisplatin on the cisplatin-resistant SCC-25/CP line. The ED50 value of DAC-SCC-25 and DAC-SCC-25/CP were comparable at 6.55 µM and 6.96 µM, respectively (Figure 1A-D).

Additionally, we determined the effect of decitabine pre-treatment on cisplatin-mediated apoptosis. Figure 1E illustrates caspase 3/7 activity in SCC-25 and SCC-25/CP cells after cisplatin treatment, with higher activity denoting increased apoptosis. When compared to SCC-25 cells, SCC-25/CP had significantly lower apoptotic activity in response to cisplatin treatment at dose ranges of 3.6–100 µM, indicating resistance to cisplatin. Pre-treatment with decitabine restored the apoptotic activity of cisplatin on cisplatin-resistant cells, such that DAC-SCC-25/CP cells had significantly higher apoptotic activity than SCC-25/CP cells in response to cisplatin treatment. Decitabine pre-treatment also enhanced the apoptotic effects of cisplatin on cisplatin-sensitive SCC-25 cells. DAC-SCC-25 cells had significantly higher apoptotic activity than SCC-25 cells, indicating an additional apoptotic benefit of decitabine treatment even when cancer cells are sensitive to cisplatin.

### Decitabine and cisplatin combination treatment inhibited growth of cisplatin-resistant SCC in a mouse model

**Figure 2:**
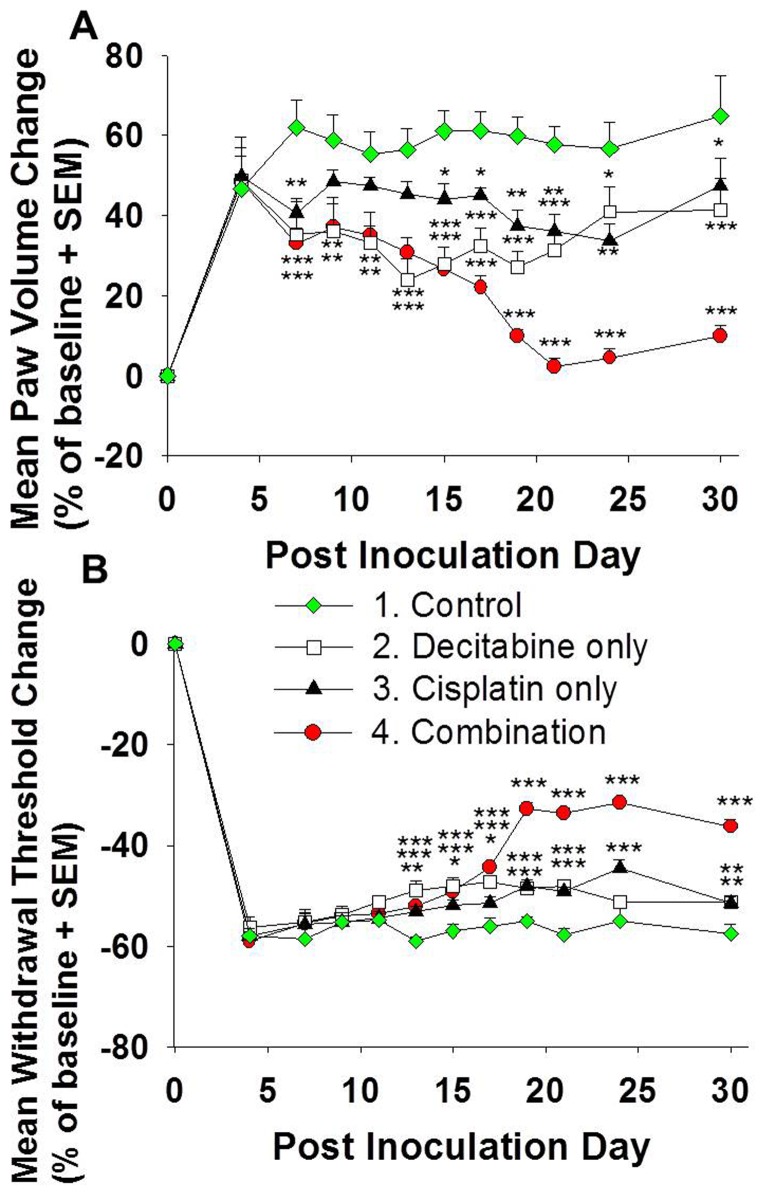
Combination treatment with decitabine and cisplatin results in significant anti-tumor and antinociceptive effects. (A) Combination treatment of decitabine and cisplatin produces a stronger anti-tumor effect in the preclinical model than either drug alone. Paw volume change from baseline (day 0 prior to cancer inoculation) are shown. Treatment with either decitabine or cisplatin alone produces a minor, non-sustained reduction in paw volume. Combination treatment with decitabine and cisplatin, however, produces sustained anti-tumor activity even after cessation of cisplatin treatment on PID 21. (B) The graph shows percent change in mechanical threshold from baseline. Mechanical threshold of mice treated with decitabine, cisplatin and combination treatment was significantly higher than the control groups on indicated days, signifying lower mechanical allodynia (*p<.05, **p<.01, ***p <.001, Two-way RM ANOVA, Holm-Sidak test, see Table 2).

To determine whether including decitabine in the chemotherapy regimen augments the anti-tumor effect of cisplatin in cisplatin-resistant HNSCC *in vivo*, we created a mouse HNSCC model by inoculating SCC-25/CP cells into the right hind paw of BALB/c athymic mice. We chose the hind paw as the xenograft site because tumor volume and mechanical hypersensitivity could be reliably quantified at this site. SCC-25/CP inoculation resulted in tumor growth in the hind paw, represented by increased paw volume (Figure 2A) starting on PID 4. Decitabine treatment on PID 7 and 9 resulted in inhibition of tumor growth; however, this effect was not significant at the end of the experiment on PID 30, indicating that the effect of decitabine treatment alone could not be sustained. Cisplatin-only treatment also resulted in tumor growth inhibition, but the effect was not sustained after drug treatment was stopped on PID 21. Paw volume in the cisplatin-only group was not significantly different from the control group on PID 30. Combination treatment with decitabine and cisplatin was most effective in inhibiting tumor growth, and this inhibitory effect was sustained until PID 30. The mean paw volume change for the combination group was 10% on PID 30, compared to 65% in the control group. Body weight was not significantly different among all four groups during weekly measurements, indicating that the drug doses used did not cause cachexia (data not shown).

### Decitabine and cisplatin combination treatment resulted in the least mechanical allodynia in a cisplatin-resistant SCC mouse model

In addition to tumor growth inhibition we also determined the effects of drug treatment on cancer-induced pain. HNSCC patients most frequently complain of orofacial functional restriction due to pain [10]; we therefore quantified the effect of drug treatment on mechanical allodynia in our preclinical model. Figure 2B depicts the change in mechanical withdrawal threshold from baseline, with a decrease from baseline signifying increased mechanical allodynia. SCC-25/CP tumor growth resulted in increased mechanical allodynia, with a 58% decrease on PID 30 compared to baseline (4.22 g at baseline). When compared to the control group, combination treatment with decitabine and cisplatin resulted in the most significant reduction in mechanical allodynia. The mechanical threshold of the combination group only decreased by 36% on PID 30 (2.69 g from 4.23 g at baseline).

### Methylation profiles were different between cisplatin-responsive and cisplatin-unresponsive HNSCC

**Figure 3:**
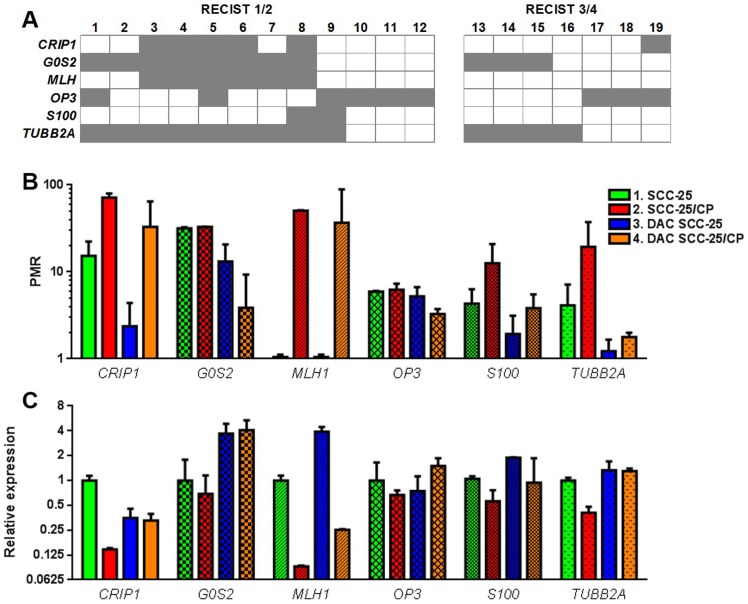
Methylation profiles were different between cisplatin-responsive and cisplatin-unresponsive HNSCC cancer tissues and cell lines. (A) A matrix of methylation profiles in cisplatin-unresponsive HNSCC tumors (RECIST 1 or 2) and cisplatin-responsive tumors (RECIST 3 or 4) was created using PMR = 10 as the cutoff for methylation positivity. Cisplatin-unresponsive tumors were more likely to be methylated at within the chosen gene panel (*CRIP1, G0S2, MLH1, OPN3, S100* and *TUBB2A*) than cisplatin-responsive tumors (66.7% cisplatin-unresponsive tumors vs 0% cisplatin-responsive tumors had 3 or more methylated genes). (B) The bar graph shows PMR values of SCC-25 and SCC-25/CP before and after decitabine treatment (*i.e.*, DAC-SCC-25 and DAC-SCC-25/CP) for each of the six genes. SCC-25/CP cisplatin-resistant cells had a significant hypermethylated methylation signature compared to SCC-25 cisplatin-sensitive cells. Decitabine treatment reversed methylation of SCC-25/CP cells toward a cisplatin-sensitive profile; the methylation signature of the six-gene classifier was significantly different between SCC-25/CP and DAC-SCC-25/CP cells. (C) The bar graph shows relative expression of the six genes for the two cell lines before and after decitabine treatment. SCC-25/CP cells had significantly lower expression levels for the six-gene classifier compared to SCC-25 cells. Decitabine treatment of SCC-25/CP cells (*i.e.*, DAC-SCC-25/CP) increased expression levels toward a cisplatin-sensitive expression profile. (See Tables 2 and 3 for statistical analysis.)

We obtained FFPE tissue from 19 patients with biopsy-proven HNSCC who were treated with cisplatin. All 19 patients also received radiation in addition to cisplatin chemotherapy. Patient demographics are detailed in Table 1. None of the samples were positive for HPV16 E6 mRNA as detected by PCR (results not shown). We then categorized the tumors according to RECIST criteria, with RECIST 3 or 4 being “cisplatin responsive” (n = 7) and RECIST 1 or 2 being “cisplatin unresponsive” (n = 12). We quantified methylation within the promoter region of six genes: *CRIP1, G0S2, MLH1, OPN3, S100* and *TUBB2A*. These genes have been implicated in cisplatin resistance of carcinomas other than HNSCC [11], [22], [23]. We used the calculated PMR value to classify each sample as either “positive” or “negative” for methylation, using a cutoff of 10 based on our previous publication [24]. In the Figure 3A matrix, in which samples that were positively methylated for a gene were colored grey, the cisplatin unresponsive group had more methylated samples. 8 of 12 samples in the cisplatin unresponsive group, and no samples in the cisplatin responsive group, had positive methylation of at least 50% of the genes in the gene panel.

**Table 1:** Patient Demographics.

Case #	Sex	Age	Site	TNM	Disease burden at 6 months (RECIST)
1	F	58	retromolar trigone	T2N1M0	progressed (1)
2	M	56	tongue	T4aN2bM0	progressed (1)
3	M	48	retromolar trigone	T4aN0M0	progressed (1)
4	F	20	tongue	T4aN2bM0	progressed (1)
5	M	57	tongue	T4aN2bM0	progressed (1)
6	M	67	soft palate	T3N2bM0	progressed (1)
7	M	50	base of tongue	T2N2bM0	progressed (1)
8	M	59	base of tongue	T2N2cM1	progressed (1)
9	M	34	tongue	T1N1M0	progressed (1)
10	F	66	tongue	T1N1M1	progressed (1)
11	F	74	upper lip	T1N1M0	stable (2)
12	M	67	tongue	T2N0M0	stable (2)
13	M	61	tonsil	T1N2bM0	complete remission (4)
14	F	62	base of tongue	T4N2M0	complete remission (4)
15	F	71	base of tongue	T1N3M0	complete remission (4)
16	M	50	tonsil	T2N2aM0	complete remission (4)
17	F	51	tongue	T2N2M0	complete remission (4)
18	M	81	floor of mouth	T2N0M0	complete remission (4)
19	M	69	floor of mouth	T4aN1M0	complete remission (4)

The same methylation trend was present *in vitro*. We compared methylation levels of the cell lines across the six genes with two separate statistical methods. Firstly we used the six genes as a single classifier (Two-way ANOVA, Tukey test, see Table 2 for statistical summary). Secondly we compared the methylation levels of the cell lines for each separate gene (Student's t test, Table 3). While methylation of the separate genes was not significantly different between SCC-25 and SCC-25/CP, the methylation signature of the whole gene panel was significantly different between the two cell lines (Table 2). The methylation signature of the entire classifier was also significantly different between SCC-25/CP and DAC-SCC-25/CP cells.

**Table 2:** Statistical Summary of Two-way ANOVA and *Post Hoc* Analyses.

	Two-way ANOVA				*Post Hoc* Analysis	
	Effects	DF	F	P	Groups	P
**Figure 2A**	Tx	3	83.473	<0.001	4 vs. 1	<0.001
	Time	11	24.079	<0.001	2 vs. 1	<0.001
	Time × Tx	33	3.372	<0.001	3 vs. 1	<0.001
**Figure 2B**	Tx	3	17.841	<0.001	4 vs. 1	<0.001
	Time	11	875.806	<0.001	2 vs. 1	0.001
	Time × Tx	33	19.748	<0.001	3 vs. 1	0.007
**Figure 3B**	Cell line	3	12.37	<0.001	1 vs. 2	<0.001
	Gene	5	7.763	<0.001	1 vs. 3	ns
	Cell line × gene	15	2.652	0.0065	2 vs. 4	<0.05
**Figure 3C**	Cell line	3	9.96	<0.001	1 vs. 2	<0.01
	Gene	5	24.34	<0.001	1 vs. 3	<0.001
	Cell line × gene	15	3.999	0.001	2 vs. 4	<0.001

**Table 3:** Statistical Summary of Individual Gene Comparisons.

	*CRIP1*	*G0S2*	*MLH1*	*OP3*	*S100*	*TUBB2A*
**Student's t tests comparing Percent of Methylated Reference**						
**SCC-25 vs. SCC-25/CP**	0.0588	0.224	<0.0001	0.7096	0.1042	0.1456
**SCC-25 vs. DAC SCC-25**	0.016	0.0747	0.4228	0.5713	0.0884	0.1728
**SCC-25/CP vs. DAC SCC-25/CP**	0.2077	0.0172	0.004	0.068	0.0872	0.1595
**Student's t tests comparing gene expression**						
**SCC-25 vs. SCC-25/CP**	0.0126	0.6061	0.0127	0.0214	0.0813	0.0007
**SCC-25 vs. DAC SCC-25**	0.0027	0.0146	0.0189	0.3702	0.0037	0.2028
**SCC-25/CP vs. DAC SCC-25/CP**	0.0222	0.0566	0.0004	0.0835	0.6253	0.0002

To determine whether promoter methylation correlated with gene expression, we quantified mRNA of the six genes in SCC-25 and SCC-25/CP cells before and after decitabine treatment (*i.e.*, DAC-SCC-25 and DAC-SCC-25/CP cells). We compared relative expression of the cell lines using SCC-25 as the reference cell line (Figure 3B). We performed statistical analyses of the expression data for each separate gene (Table 3) and for the gene classifier of six genes (Table 2). SCC-25/CP cells had significantly lower expression than SCC-25 cells in four of the six genes. Decitabine treatment changed gene expression—such that expression of the gene classifier was significantly different between non-treated and decitabine-treated cells for both SCC-25 and SCC-25/CP cells (Table 2). When we analyzed each gene separately, decitabine resulted in either promoter demethylation or increase in gene expression of *CRIP1, G0S2, MLH1*, and *S100* in SCC-25 and *CRIP1, G0S2, MLH1*, and *TUBB2A* in SCC-25/CP.

## Discussion

### Decitabine restores cisplatin sensitivity and treats cancer-induced pain

The incidence of head and neck cancer is increasing, especially in younger people [25]. Chemoradiation with cisplatin remains the mainstay of primary or adjuvant treatment in these patients. Patients who are resistant to cisplatin suffer from cancer-induced pain and poor survival. While several mechanisms for cisplatin resistance have been established, none of the reported mechanisms are reversible. In this study we hypothesized that methylation of key genes is a molecular mechanism leading to cisplatin resistance. We decided to investigate DNA methylation as a resistance mechanism because it is reversible by available drugs. We used SCC-25 and its cisplatin-resistant counterpart, SCC-25/CP, and determined that pre-treatment with the demethylating drug decitabine enhanced the anti-proliferative and apoptotic effect of cisplatin on these cell lines. In our HNSCC mouse model, combination treatment with decitabine and cisplatin produced a more robust anti-tumor effect than either drug alone. Decitabine pre-treatment *in vitro* reversed cisplatin-resistance in SCC-25/CP cells, and lowered the dose of cisplatin required to produce anti-proliferative or apoptotic effects. Interestingly, decitabine pre-treatment also lowered the dose of cisplatin required for cisplatin-sensitive SCC-25 cells. The clinical significance of our results is that decitabine could salvage patients with cisplatin-resistant tumors; moreover, for those patients who have cisplatin-sensitive tumors, decitabine could lower the cisplatin dose required, allowing for reduced toxicity.

Previous studies have explored the effectiveness of epigenetic therapy in rescuing cisplatin resistance in other cancers. Adding hydralazine and valproate to cisplatin therapy significantly increased progression-free survival in advanced stage cervical cancer patients [26]. A phase I trial for patients with solid tumors showed that combination treatment of decitabine followed by carboplatin is safe [27]. A phase II study adding valproate and hydralazine to the same schedule of chemotherapy on which patients with solid cancers were progressing showed clinical benefit in 12 of 15 (80%) patients [28]. At the same time there have been studies adding demethylating agents to platinum-based chemotherapy with negative results. A phase II trial randomized ovarian cancer patients progressing 6–12 months after previous platinum therapy to one of two groups: one group would receive decitabine with carboplatin, and the second group would receive carboplatin alone. However the study closed after an interim analysis showed that the combination group had lack of efficacy and poor treatment deliverability [29]. Our dose scheduling of decitabine and cisplatin is based on previous work in ovarian and colon carcinoma [23] showing that multiple doses of decitabine are required prior to cisplatin administration to maximally sensitize xenografts to cisplatin.

In addition to reduced survival, head and neck cancer patients have significant function-limiting pain, which is either cancer-induced or treatment-induced. While survival and pain seem like unrelated issues, a recent randomized clinical trial shows that aggressive pain management in advanced-stage cancer patients significantly improves quality of life and increases survival [30]. Peripheral neuropathy is a major toxic side effect of cisplatin and contributes to pain [31]. The behavioral assay that we used on our preclinical model detects both cancer-induced pain and neuropathic pain. We showed that combination therapy of decitabine and cisplatin resulted in significantly reduced mechanical pain. While nociception in our preclinical model was likely cancer-induced, decitabine treatment potentially reduces the required cisplatin dose, thus minimizing peripheral neuropathy.

### Methylation classifier for cisplatin resistance

HNSCC survival has not dramatically improved, even in an era of burgeoning personalized medicine, for two reasons. The first reason is that no effective treatment has been developed to combat cisplatin resistance. The second is that there is no effective marker to predict cisplatin responsiveness. Therefore, in addition to re-purposing decitabine as a drug to rescue cisplatin-resistance, we developed a methylation and expression classifier that could differentiate between cisplatin-responsive and cisplatin-unresponsive HNSCC. The classifier must have the additional ability to predict decitabine efficacy in the setting of cisplatin-resistance. Previous studies have shown that although many genes are hypermethylated and downregulated in cisplatin resistant cancer, only a small proportion of these genes are re-expressed in response to decitabine treatment [32]. In developing a classifier that could potentially be used to monitor decitabine efficacy in patients with cisplatin-unresponsive HNSCC, we targeted genes that (1) are hypermethylated in cisplatin-unresponsive tumors and (2) can be re-expressed *in vitro* with decitabine treatment. We therefore combined methylation data from patient tumor tissues and cell lines following decitabine treatment to converge on six genes (*CRIP1, G0S2, MLH1, OPN3, S100* and *TUBB2A*) as the classifier. These six genes have been shown in previous studies to be hypermethylated in cisplatin-resistant cell lines [11], but their methylation status in cancer tissue of HNSCC patients has not been quantified. One of the six genes, *MLH1*, has been shown to directly confer cisplatin sensitivity when re-expressed in ovarian cancer cells *in vitro* [23], [32]. We showed that methylation of the six genes was higher in cisplatin-unresponsive tumors (RECIST 1/2) than cisplatin-responsive tumors (RECIST 3/4) of HNSCC patients. Moreover, when we assembled the six genes into a classifier and used the criterion of positive methylation in three or more genes to categorize cisplatin sensitivity, we could differentiate cisplatin-responsive from cisplatin-unresponsive tumors with a sensitivity of 67% and a specificity of 100%. The modest sensitivity is in part due to our small sample size—it was difficult to obtain initial biopsy samples with adequate tissue for DNA extraction. Another limitation is that we could not correlate methylation with gene expression, since we had obtained limited quantities of formalin-fixed, paraffin-embedded tissue that could not be used to reliably quantify mRNA or perform immunohistochemical staining. We therefore used our cell lines to determine whether there was a functional correlation between gene methylation status and expression levels. We showed that the cisplatin-resistant (SCC-25/CP) cells had a significantly different methylation signature of the six gene classifier compared to cisplatin-sensitive (SCC-25) cells. We then treated the cell lines with decitabine. We showed that decitabine treatment produced either a significant decrease in methylation or increase in gene expression in four of the six genes for both SCC-25 and SCC-25/CP. When all six genes were used as a single classifier, we showed statistically significant differences between the decitabine-treated and non-treated cell lines. Therefore the six genes responded to decitabine treatment and could potentially be used to monitor decitabine efficacy in cisplatin-resistant HNSCC.

Chemotherapy with radiation is typically used for HNSCC, as adjuvant post-surgical treatment for tumors with worrisome features [33], or as primary treatment for advanced stage disease. Cisplatin remains the most frequently used and most effective chemotherapy, as it is thought to act synergistically with ionizing radiation by enhancing the formation of cluster damage to DNA [34]. One perceived limitation of the study is that the preclinical model did not accurately replicate HNSCC treatment, since radiation was not included in the treatment scheme. Our rationale for not including radiation was to isolate the effects of cisplatin on the cancer cells and to eliminate any synergy between cisplatin and radiation.

In summary, our study establishes methylation as a mechanism of cisplatin resistance, and pre-treatment with a demethylating drug as a possible strategy to reduce cisplatin resistance. While the role of gene methylation on cisplatin sensitivity has been explored *in vitro* [11], our study uses a preclinical cisplatin-resistant HNSCC model to determine the effect of decitabine on proliferation and pain. Furthermore, we utilize cancer tissues from HNSCC patients to create a classifier for cisplatin-resistance. Despite limited sensitivity of the classifier due to small sample size, we show in the cell lines that decitabine treatment reverses methylation and increases expression of genes within the classifier. Our current results lay the groundwork for future studies focused on demethylation therapy for cisplatin resistance and methylation markers as a method to identify patients with cisplatin resistance.
